# Patients' Ability to Self‐Manage Their Surgical Wound to Prevent Wound Complications: A Cross‐Sectional Study

**DOI:** 10.1111/jan.16644

**Published:** 2024-11-25

**Authors:** Hannah Groenen, Nathan Bontekoning, Susan Van Dieren, Ricardo G. Orsini, Marja A. Boermeester, Georgia Tobiano, Anne M. Eskes

**Affiliations:** ^1^ Amsterdam UMC Location University of Amsterdam, Department of Surgery Amsterdam The Netherlands; ^2^ Amsterdam Gastroenterology Endocrinology & Metabolism Amsterdam The Netherlands; ^3^ Department of Surgery Maastricht University Medical Center+ Maastricht The Netherlands; ^4^ National Health and Medical Research Council Centre of Research Excellence in Wiser Wound Care Griffith University Southport Queensland Australia; ^5^ Nursing and Midwifery Education and Research Unit Gold Coast University Hospital, Gold Coast Hospital and Health Service Southport Queensland Australia; ^6^ School of Nursing and Midwifery Griffith University Gold Coast Queensland Australia

**Keywords:** discharge education, postoperative wound complications, surgical site infection, surgical wound care

## Abstract

**Aims:**

To provide insights into postdischarge patients' experiences and preferences regarding surgical wound care education at discharge in the Netherlands.

**Background:**

Patient involvement in wound care practices postdischarge is beneficial for the prevention of surgical site infections and has become an essential component in reducing the burden on healthcare systems. Surgical wound care discharge education is crucial to achieve this.

**Methods:**

A cross‐sectional survey was conducted using the 18‐item Surgical Wounds And Patient Participation Questionnaire on patients who underwent surgery between January and May 2022. The survey was administered 2 weeks postoperatively.

**Results:**

In total, 213 patients completed the survey. Most patients preferred verbal instructions about their surgical wound care (*n* = 146; 84.9%) at the time of discharge, with 52.3% indicating a preference for multiple forms of information. Approximately three‐quarters of patients (*n* = 99; 76.7%) responded that they were able to successfully take care of their wounds at home and 16.3% indicated wound self‐care inability.

**Conclusion:**

Our study highlighted patients' preferences for verbal instructions about their surgical wound care at the time of discharge. Notably, half of the patients expressed a desire for multiple forms of information. Furthermore, we identified gaps in the information provided to patients, indicating areas for improvement in discharge communication.

**Implications:**

The identified gaps in surgical wound care discharge education offer opportunities to enhance in‐hospital education by aligning it more closely with patient preferences and providing education of topics often missed. This could ultimately improve their ability to self‐manage their surgical wound at home. Future research should delve deeper into understanding the factors influencing patients' ability to self‐manage their wounds.

**Impact:**

- Despite the acknowledged importance of patient education on surgical wound care, there is limited literature regarding patients' experiences and preferences in this area.
- The findings of this study highlight patients' preferences for verbal instructions and reveal gaps in the information provided to patients about surgical wound care at the time of discharge.
- The identified gaps in information provided to surgical patients offer opportunities to enhance in‐hospital education by healthcare professionals.

**Reporting Method:**

We adhered to the STROBE guideline.

**Patient or Public Contribution:**

No patient or public contribution.

**Summary**

- What Does this Paper Contribute to the Wider Global Clinical Community?
  ○ The findings of this study are crucial for improving surgical wound care education at discharge, an essential component in enhancing healthcare efficiency and reducing the burden on healthcare systems.
  ○ Most patients preferred to receive verbal instructions about their surgical wound care from medical or nursing staff at the time of discharge, with more than half of patients preferring multiple forms of information.
  ○ The identified gaps in surgical wound care discharge education offer opportunities to enhance in‐hospital education by aligning it more closely with patient preferences and providing education on topics often missed.

## 1 Introduction

Surgical site infection (SSI) is the most common postoperative complication, associated with increased morbidity, mortality, prolonged hospital stay and higher healthcare costs (Gillespie et al. 2021; Badia et al. 2017; Pinto et al. 2016). The incidence of SSI overall is estimated at 2%, but there is great variability across procedure types (Griškevičienė 2013). A 2021 systematic review and meta‐analysis reported a global 30‐day cumulative incidence of 11% following general surgery, with the highest rates observed in hepatobiliary procedures at 19% (Gillespie et al. 2021). In the Netherlands, SSI rates range from 0.4% in primary half‐knee prothesis to 16.5% in open colon surgery (PREZIES 2022). SSI impose a substantial financial burden, with the attributable costs per SSI reported at €14,084 for colectomy and €21,569 for total hip arthroplasty in the Netherlands (Koek et al. 2019). In the United States, the average cost per patient case is $20,785, contributing to an estimated annual healthcare burden of approximately $1.6 billion (Zimlichman et al. 2013). Patient involvement in postoperative care has recently become an essential component in enhancing healthcare efficiency and reducing the burden on healthcare systems (Yun et al. 2020). Since the majority of SSI occur postdischarge, patient participation in self‐management of wound care practices can be beneficial in the prevention of SSI (Tartari et al. 2017). To convey the knowledge and skills for patients to participate in wound care management at home, timely education on surgical wound care during hospital admission is crucial (Kang et al. 2018).

Currently, the shift from in‐hospital to postdischarge surgical care still poses challenges, including variations in discharge education. Patients state that managing their surgical wounds after discharge is difficult due to inadequate education during the postoperative period (Andersson et al. 2010). Additionally, patients who have experienced SSI report deficiencies in discharge education, self‐monitoring of wounds at home and communication with healthcare providers (Sanger et al. 2014). Notably, patients who receive more comprehensive discharge information than anticipated report significantly fewer wound complications (Koivisto et al. 2020). In addition to minimising wound complications, enhancing patients' self‐management also results in improved knowledge about their health, heightened satisfaction, physical and psychological quality of life (Park et al. 2018).

Despite the acknowledged importance of patient education on surgical wound care, there is limited literature regarding patients' experiences and preferences in this area (Kang et al. 2018). To address this gap, an 18‐item Surgical Wounds And Patient Participation Questionnaire (SWAPP‐Q) was developed (Tobiano, Chaboyer et al. 2023). The findings from the survey conducted in Australia indicated that patients in Australia prefer verbal and written discharge information delivered by medical and nursing staff (Tobiano, Walker et al. 2023). They highlighted the critical need to include shared decision‐making, patient participation and pain discussions, into discharge education to enhance patients’ ability to manage their wounds once home.

While providing important insights into surgical wound care education, the transferability of these findings to patients in the Netherlands remains uncertain. This study aims to address this by presenting the SWAPP‐Q survey on patient experiences and preferences in surgical wound care education at discharge within the Dutch context. Through this study, we aim to provide valuable insights for refining and optimising postoperative care practices in similar settings.

## 2 Methods

This study adheres to the Strengthening the Reporting of Observational Studies in Epidemiology (STROBE) Statement: guidelines for reporting observational studies (von Elm et al. 2008).

### 2.1 Study Design, Setting and Sampling

A cross‐sectional survey design was used. We included a convenience sample of patients who underwent surgery of any type at two sites of a large tertiary academic medical centre in Amsterdam, the Netherlands, between January and May 2022. Together, these two sites perform approximately 19,000 surgeries annually, with surgical subspecialties distributed between them. Although the subspecialties vary by site, both locations operate as a single, unified hospital. The surgical wards involved in the study primarily care for patients recovering from (oncological) gastro‐intestinal, trauma, orthopaedic, vascular, plastic, urological and oral–maxillofacial surgeries.

### 2.2 Inclusion and Exclusion Criteria

Inclusion criteria comprised adult patients who underwent either elective or emergency surgical procedures (including both open and laparoscopic surgeries), received a surgical incision (surgical wound), were admitted for at least one night, were competent to give consent for research participation and were available for telephone interview 2 weeks after surgery. Patients were excluded if they declined or were unable to provide consent, or if they were unable to understand Dutch.

### 2.3 Data Collection

Eligible patients were approached in person by a nursing student, supervised by a trained nurse researcher and received written and oral explanation of the study. Patients willing to participate were asked to provide written and oral consent. Contact details, demographic and clinical data were collected. Two weeks after surgery, consenting participants were contacted over telephone by a nursing student to conduct the survey. Two follow‐up phone calls were made if they did not answer the first time. Participants' demographic, clinical and survey data were recorded in a web‐based data management system (Castor) and coded using a unique identification number.

### 2.4 Instruments

The SWAPP‐Q survey used in this study investigates patients' experiences and preferences in surgical wound care discharge education and participation in wound care decisions (Tobiano, Chaboyer et al. 2023). The survey contained four themes with 18 items: (1) Wound care discharge education (10 items); (2) Participation in wound care decisions (3 items); (3) Patients' ability to manage their surgical wound to prevent wound complications (1 item) and (4) Preferences for discharge education delivery (4 items). The complete survey can be found in Data S1. Response options varied from yes/no/not applicable, multiple response options and 5‐point Likert scale response options (strongly disagree–strongly agree).

### 2.5 Translation and Validation

The survey was translated from English into Dutch using the forward–backward method (Lee et al. 2019). The forward translation was conducted by a native Dutch speaker proficient in English, and subsequent backward translation was performed by a native English speaker (CD) with a good proficiency in the Dutch language. The translated survey has only undergone content validity testing and has not been subjected to the additional statistical tests necessary for comprehensive questionnaire validation (Terwee et al. 2007).

### 2.6 Data Analysis

Descriptive statistics were calculated, including numbers and percentages for categorical variables, means and standard deviations or median and interquartile ranges for continuous variables, depending on the distribution of the data. Descriptive analysis was performed on survey items with multiresponse options. To assess the distribution of the data, we employed histogram plots and boxplots. Patients with missing responses and those still hospitalised at the time of the survey were excluded from the analysis, as they likely had not yet received discharge education on surgical wound care. For all questions that included the ‘Not Applicable’ response option—indicating that the question was irrelevant to the patient's postsurgery care—these responses were treated as valid but were excluded from the analysis for that specific question. Consequently, percentages for ‘Yes’ and ‘No’ responses were calculated based only on the relevant responses. All analyses were done in R, version 4.2.1 (R Foundation for Statistical Computing, Vienna, Austria).

### 2.7 Ethical Considerations

The Institutional Review Board of the Amsterdam University Medical Centers, location AMC, decided that ethical approval of this study was not required as per the Medical Research Involving Human Subjects Act (Reference W21_440 # 21.489) because the participants were not subjected to interventions or asked to follow any study‐related procedures. Participation was voluntary and the participant could withdraw at any moment. Patients gave written and verbal informed consent. All data were collected, analysed and reported anonymously.

## 3 Results

### 3.1 Characteristics of the Sample

A total of 213 surgical patients completed the survey, of whom 172 were included in the analysis because they had been discharged from the hospital. Only discharged patients were included to ensure that they had received discharge education on surgical wound care, as patients still hospitalised at the time of the survey may not have been fully informed. Baseline demographic characteristics are presented in Table 1. Mean age was 56.7 years (standard deviation 15.8), and most patients had a vocational education or higher qualifications (74.7%). Patients underwent different types of surgery.

**TABLE 1:** Baseline and surgical characteristics.

Characteristics	Total (*n* = 172)
Age, years (mean, SD)	56.7 (15.8)
Missing	0
Sex (%)	
Female	81 (47.1)
Male	91 (52.9)
Missing	0
Education level (%)	
No education	3 (1.8)
Primary education	8 (4.7)
Secondary education	32 (18.8)
Vocational education	47 (27.6)
Bachelor education	55 (32.4)
University education	25 (14.7)
Missing	2 (1.2)
Surgery type (%)	
Orthopaedic	44 (25.6)
Trauma	37 (21.5)
Gastro‐intestinal	31 (18.0)
Gastro‐intestinal oncologic	26 (15.1)
Vascular	23 (13.4)
Plastic	9 (5.2)
Oral and maxillofacial	1 (0.6)
Urology	1 (0.6)
Missing	0
Wound location (%)	
Leg/Hip/Ankle/Foot	67 (39.0)
Abdomen	63 (36.6)
Arm/Shoulder/Hand	13 (7.6)
Back	7 (4.1)
Groin area	10 (5.8)
Head/Scalp/Face/Neck	5 (2.9)
Chest	3 (1.7)
Abdomen and chest	2 (1.2)
Arm, leg and head	1 (0.6)
Abdomen and groin area	1 (0.6)
Missing	0

Abbreviation: SD, standard deviation.

### 3.2 Patient Experiences in Surgical Wound Care Education

Table 2 presents the detailed survey responses corresponding to Theme 1, patient experiences of surgical wound care education. More than half of the patients received instructions on how to clean their wound (*n* = 80; 60.6%), what wound dressing to use at home (*n* = 77; 71.3%) or when and how stitches are removed (*n* = 77; 60.6%). Additionally, most patients received instructions on what activities to avoid during wound healing (*n* = 124; 75.6%), while half of patients received instructions specifically addressing the signs of wound infection (*n* = 86; 52.8%). Patients were frequently provided with logistic instructions, which included information about arrangements made for follow‐up appointments (*n* = 144; 85.7%) and who to contact in case of concerns regarding their surgical wound (*n* = 144; 86.2%). Most information was communicated verbally and the majority of patients (*n* = 138; 85.2%) had the opportunity to pose questions about their wound care prior to discharge. Furthermore, almost one‐third of patients (*n* = 55; 32.0%) received multiple forms of information. However, 26 patients (15.1%) reported not receiving any information.

**TABLE 2:** Theme 1 survey responses: Wound care discharge education (*n* = 172).

I was given instructions about …^a^	Yes (*n* (%))	No (*n* (%))	Total respondents excluding NA (*n*)
Who to contact if I had concerns about my surgical wound or about caring for the wound	144 (86.2)	23 (13.8)	167
Arrangements made for follow‐up appointments	144 (85.7)	24 (14.3)	168
What activities I should avoid during wound healing	124 (75.6)	40 (24.4)	164
The signs of infection in the wound	86 (52.8)	77 (47.2)	163
How the wound should be cleaned	80 (60.6)	52 (39.4)	132
When and how stitches (or ‘tape’/steri‐strips or staples) are removed	77 (60.6)	50 (39.4)	127
The wound dressing I would use at home	77 (71.3)	31 (28.7)	108

Before I was discharged from hospital, I had the opportunity to ask questions about how to care for my surgical wound (total respondents excluding ‘not applicable’: *n* = 162)
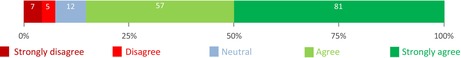

How did you receive information about caring for your surgical wound?	Yes (*n* (%))
Verbal instructions with questions and answers	101 (58.7)
Printed material such as a brochure, checklist or information sheet	68 (39.5)
I did not receive any information	26 (15.1)
Verbal instructions with no discussion	23 (13.4)
Other	13 (7.6)
Teaching by using visual materials such as pictures or models	4 (2.3)
Online reading material	0 (0.0)
Online audio/visual material	0 (0.0)
Patients that selected more than one answer option^b^	55 (32.0)

Abbreviation: NA, not applicable.

^a^ The relevant response counts differ per question due to the exclusion of the NA responses. Percentages for ‘Yes’ and ‘No’ responses were calculated based only on the relevant responses.

^b^ Patients could select more than one answer option. Therefore, the number of patients who selected multiple answer options is also shown.

### 3.3 Participation in Wound Care Decisions

Data from Theme 2 (Table 3) show that approximately half of the patients (*n* = 86; 54.1%) were engaged in discussions about their treatment options in relation to wound care with medical and/or nursing staff. Moreover, most patients (*n* = 130; 79.8%) stated that pain management options for wound‐related pain were discussed. However, only 63 patients (42.0%) were invited to participate in the decision‐making process concerning their wound care.

**TABLE 3:** Theme 2 survey responses: participation in wound care decisions (*n* = 172).

	Yes (*n* (%))	No (*n* (%))	Total respondents excluding NA (*n*)
Have doctors and/or nurses discussed pain management options for wound‐related pain?	130 (79.8)	33 (20.2)	163
Have doctors and/or nurses discussed your treatment options in relation to wound care?	86 (54.1)	73 (45.9)	159
Were you invited to participate in decision‐making about your wound care?	63 (42.0)	87 (58.0)	150

*Note:* The relevant response counts differ per question due to the exclusion of the N/A responses. Percentages for ‘Yes’ and ‘No’ responses were calculated based only on the relevant responses.

Abbreviation: NA, not applicable.

### 3.4 Patients' Ability to Manage Their Surgical Wound

**FIGURE 1:**
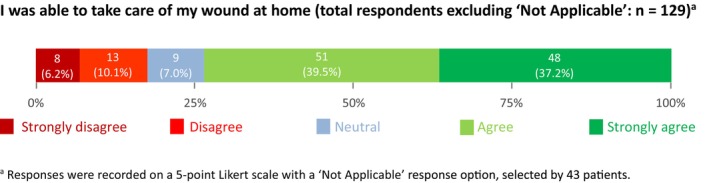
Theme 3 survey responses: patients' ability to manage their surgical wound to prevent wound complications (*n* = 172).

In Theme 3, just over three‐quarters of patients (*n* = 99; 76.7%) responded that they were able to successfully take care of their wounds at home (Figure 1). Twenty‐one patients (16.3%) stated that they were unable to successfully manage care of their wounds. The ability to self‐care varied by surgery type (Data S2). In addition, Data S3 highlights the variation by wound location, showing that patients with wounds in the groin area reported a low ability to self‐manage their wound, with only three patients (33.3%) stating they could do so.

### 3.5 Patient Preferences for Surgical Wound Care Education

Theme 4 focuses on understanding patient preferences for surgical wound care (Table 4). More than half of patients (*n* = 90; 52.3%) preferred multiple forms of education. The majority of patients (*n* = 146; 84.9%) preferred receiving verbal instructions, mostly with the possibility of posing questions and answers (*n* = 130; 75.6%), about their surgical wound care from the medical (*n* = 103; 59.9%) or nursing staff (*n* = 106; 61.6%) at the time of discharge (*n* = 119; 64.0%). Furthermore, most patients (*n* = 128; 74.4%) reported that they prefer a physical consultation for their follow‐up appointment.

**TABLE 4:** Theme 4 survey responses: Patient preferences (*n* = 172).

How would you like to receive information about caring for your surgical wound?	*n* (%)
Verbal instructions with questions and answers	130 (75.6)
Printed material such as a brochure, checklist or information sheet	89 (51.7)
Teaching by using visual materials such as pictures or models	19 (11.0)
Online reading material	10 (5.8)
Online audio/visual material	7 (4.1)
Verbal instructions with no discussion	18 (10.5)
Other	7 (4.1)
Patients that selected two answer options^a^, ^b^	75 (43.6)
Patients that selected more than two answer options^a^	15 (8.7)

How do you prefer to get your information about caring for your wound?	*n* (%)
Nursing staff	106 (61.6)
From your doctor or medical specialist includes general practitioner (GP)	103 (59.9)
Don't mind	18 (10.5)
Information leaflet	7 (0.4)
Online search	3 (1.7)
Pharmacist hospital	2 (1.2)
Family, friends	0 (0.0)
Carer	0 (0.0)
Other patients	0 (0.0)
Patients that selected more than one answer option^a^	62 (36.0)

When would you have preferred to have received information about managing your wound?	*n* (%)
At discharge	119 (64.0)
24–36 h after surgery	50 (29.0)
During my last preoperative visit to the surgeon/specialist	32 (18.6)
Other	13 (7.6)
Patients that selected more than one answer option^a^	35 (20.3)

I would prefer to have my follow‐up appointment	*n* (%)
As a hospital outpatient visit	99 (57.6)
By visiting a medical specialist or wound care nurse	29 (16.9)
As a telephone consult	34 (19.8)
Other	10 (5.8)

^a^ Patients could select more than one answer option. Therefore, the number of patients who selected multiple answer options is also shown.

^b^ Two patients selected both ‘verbal instructions with questions and answers’ and ‘verbal instructions with no discussion’.

## 4 Discussion

This study investigated patient experiences of, and preferences for, surgical wound care discharge education in the Netherlands, using the SWAPP‐Q survey. Most patients preferred to receive verbal instructions about their surgical wound care from the medical or nursing staff at the time of discharge, with more than half of patients preferring multiple forms of information. Wound care instructions at discharge often included information regarding wound dressings, what activities to avoid during wound healing, contact details for concerns and arrangements for follow‐up appointments. However, 15.1% of patients reported that they did not receive any information. Despite this, slightly over three‐quarters of patients stated that they were able to successfully take care of their wounds once home.

While we identified the same patient preferences regarding surgical wound care education as the most important, similar to those reported in Australia, these items received lower overall scores (Tobiano, Walker et al. 2023). This suggests that although Dutch patients acknowledge the same aspects of information provision as important, they may not feel as strongly about these preferences. Furthermore, our findings present notable differences in patient experiences. We observed a lower percentage of patients reporting they were able to self‐manage their wounds (76.7% compared to 90.4% in Australia). Consequently, we refrained from conducting a multiple logistic regression to identify predictors of patients' ability to manage their surgical wounds at home. The model would likely yield imprecise results, inadequate for practical clinical application. The Australian study found an association between patient participation in shared decision‐making in wound care and pain management and their perceived ability to self‐manage their wound. However, the statistically significant predictors identified in their multiple logistic regression analysis have notably wide confidence intervals, which indicates a high level of uncertainty or imprecision. Patient participation in activities such as shared decision‐making is known to enhance self‐management in chronic wound patients (Eldh 2019; Brown 2020). However, in our study, this participation was infrequently reported, a finding that aligns with existing literature on patient involvement in surgical wound care (Kang et al. 2018). Ultimately, this points to the need to enhance patient participation when Dutch patients are still hospitalised to reap the benefits of this approach to care.

As mentioned above, three‐quarters of the patients in our cohort reported the ability to self‐manage their wounds at home, while 16.3% of patients explicitly stated their inability to do so. For some patients, however, this question may have been irrelevant, particularly for those whose wounds were located in areas of the body requiring assistance for management, patients admitted to a rehabilitation centre or those receiving home care postdischarge. These patients could respond with ‘Not Applicable’, which we treated as valid but excluded from the analysis. In our cohort, 43 patients selected this option. Interestingly, this response option was not available in the Australian study, potentially introducing selection bias into their findings (Tobiano, Walker et al. 2023). Unfortunately, specific data on the number of patients admitted to a rehabilitation centre or receiving home care within our cohort are not available. Furthermore, the existing literature lacks clarity on the actual number of patients receiving surgical wound care in home care settings in the Netherlands.

### 4.1 Limitations

The current study has some limitations. First, we did not collect data on which patients received home care. Knowing this could partly explain the proportion of patients who deemed the self‐management question not applicable. We opted to exclude these responses from the analysis, resulting in a reduced sample size. Including these patients in the analysis, however, would have been problematic, as this would affect the validity of the results leading to selection bias. Second, the translated SWAPP‐Q survey has only undergone content validity testing and was not subjected to other statistical tests required for full questionnaire validation (Terwee et al. 2007). However, it is important to note that this survey is intended for exploratory use in this study. Third, the survey methodology introduces the potential for inherent subjectivity as patients are required to self‐score their ability to manage their surgical wounds, which could lead to biases in reporting. Last, the survey was administered at a single tertiary centre with two locations, potentially limiting the generalisability of our study findings. However, we included patients from a variety of different wards to minimise this limitation and enhance the broader applicability of our results.

## 5 Conclusion

Our study contributes valuable insights into patients' experiences and preferences regarding surgical wound care discharge education in the Netherlands. The identified gaps offer opportunities to enhance in‐hospital education by aligning it more closely with patient preferences and providing education of topics often missed, ultimately improving their ability to self‐manage their surgical wound at home. Future research should delve deeper into understanding the factors influencing patients' ability to self‐manage their wounds, focusing exclusively on patients not receiving surgical wound care in the home care setting.

## Author Contributions

Hannah Groenen and Anne M. Eskes were responsible for the study conception, the study design and interpretation of data. Hannah Groenen was responsible for the data curation and formal analysis. Susan Van Dieren contributed to the formal analysis. Hannah Groenen was responsible for drafting the manuscript. All authors (Nathan Bontekoning, Susan Van Dieren, Ricardo G. Orsini, Marja A. Boermeester, Georgia Tobiano and Anne M. Eskes) revised the manuscript critically for important intellectual content. All authors gave final approval of the version to be published. All authors have participated sufficiently in the work to take public responsibility for appropriate portions of the content. Hannah Groenen and Anne M. Eskes agreed to be accountable for all aspects of the work in ensuring that questions related to the accuracy or integrity of any part of the work are appropriately investigated and resolved. The authors have checked to make sure that the submission conforms as applicable to the Journal's statistical guidelines. There is a statistician on the author team: Susan Van Dieren, e‐mail: s.vandieren@amsterdamumc.nl. The authors affirm that the methods used in the data analyses are suitably applied to the data within the study design and context, and the statistical findings have been implemented and interpreted correctly. The authors agree to take responsibility for ensuring that the choice of statistical approach is appropriate and is conducted and interpreted correctly as a condition to submit to the Journal.

## Conflicts of Interest

MAB reported receiving institutional grants from J&J and 3M; and being a speaker and/or instructor for J&J, 3M, BD, Gore, Smith & Nephew, TelaBio, Angiodynamics, GDM, Medtronic and Molnlycke outside the submitted work. AME received a European Wound Management grant outside the submitted work. No other disclosures (including the use of AI and AI‐assisted technologies) were reported. All other authors report no conflicts of interest.

## Peer Review

The peer review history for this article is available at https://www.webofscience.com/api/gateway/wos/peer‐review/10.1111/jan.16644.

## Supporting information


Data S1.

Data S2.

Data S3.

## Data Availability

Data sharing is considered upon reasonable request.
