# Supplementary material for: Patients' Ability to Self‐Manage Their Surgical Wound to Prevent Wound Complications: A Cross‐Sectional Study
Source: J Adv Nurs. 2024 Nov 25;81(7):4208–15. doi: 10.1111/jan.16644 (PMC12159385; doi:10.1111/jan.16644)
Supplement: Supplementary file 1 — Data S1. [file JAN-81-4208-s002.docx]

# Supplementary file 1: The Surgical Wounds And Patient Participation Questionnaire (SWAPP-Q) survey

**1. I was able to take care of my surgical wound at home**

- Strongly disagree
- Disagree
- Neutral
- Agree
- Strongly Agree
- Not applicable

**2. In relation to your most recent surgical procedure, how was your surgical wound closed?**

*You may select more than answer:*

- Stitches
- Staples
- Tape/steri-strips?
- Adhesive glue
- Wound not closed
- Unsure
- Other, please specify ________________

**3. Did the medical staff and/or nursing staff discuss with you your treatment options in relation to wound care?**

- Yes
- No
- Not applicable

**4. Did the medical staff and/or nursing staff discuss with you your pain management options for wound related pain?**

- Yes
- No
- Not applicable

**5. Were you invited to share in the decision-making related to your wound care?**

- Yes
- No
- Not applicable

**6. Before I was discharged from hospital, I had the opportunity to ask questions about how to care for my surgical wound**

- Strongly disagree
- Disagree
- Neutral
- Agree
- Strongly Agree
- Not applicable

**We would like to know what you were told about caring for your surgical wound.**

| **I was given instructions about:** | | Yes | No | Not applicable |
| --- | --- | --- | --- | --- |
| **7.** | How the wound should be cleaned |  |  |  |
| **8.** | The wound dressing I would use at home |  |  |  |
| **9.** | The signs of infection in the wound |  |  |  |
| **10.** | Who to contact if I had concerns about my surgical wound or about caring for the wound |  |  |  |
| **11.** | What activities I should avoid during wound healing |  |  |  |
| **12.** | When and how stitches (or ‘tape’/steri-strips or staples) are removed? |  |  |  |
| **13.** | Arrangements made for follow‐up appointments |  |  |  |

**14. Where do you prefer to get your information about caring for your wound?**

*You may select more than answer:*

- From your doctor or medical specialist
- Nursing staff
- Online search
- Information leaflet
- Other patients
- Family, friends
- Carer
- Pharmacist
- Don’t mind
- Other ______________________

**15. How did you receive information about caring for your surgical wound?**

You can select more than one answer:

- Printed material such as a brochure, checklist, or information sheet
- Verbal instructions with no discussion
- Verbal instructions with questions and answers
- Teaching by using visual materials such as pictures or models
- Online reading material
- Online audio/visual material
- I did not receive any information
- Other
- If other, please specify

**16. How would you like to receive information about caring for your surgical wound?**

You can select more than one answer:

- Printed material such as a brochure, checklist, or information sheet
- Verbal instructions with no discussion
- Verbal instructions with questions and answers
- Teaching by using visual materials such as pictures or models
- Online reading material
- Online audio/visual material
- Other

**17. When would you have preferred to have received information about managing your wound?**

*You may select more than one answer:*

- During my last preoperative visit to the surgeon/specialist
- 24-36 hours after surgery
- At discharge
- Other _______________________

**18. I would prefer to have my follow-up appointment:**

- As a hospital outpatient visit
- As a telephone consult
- By visiting a medical specialist or wound care nurse
- Other _________________
