# Supplementary material for: Patients' Ability to Self‐Manage Their Surgical Wound to Prevent Wound Complications: A Cross‐Sectional Study
Source: J Adv Nurs. 2024 Nov 25;81(7):4208–15. doi: 10.1111/jan.16644 (PMC12159385; doi:10.1111/jan.16644)
Supplement: Supplementary file 3 — Data S3. [file JAN-81-4208-s003.docx]

**Supplementary file 3: Survey responses on patients’ ability to manage their surgical wound to prevent complications, stratified by wound location**

This table presents the distribution of responses to the survey question “I was able to take care of my wound at home” for different wound locations. Respondents who selected ‘Not Applicable’ were excluded from the analysis (total respondents n = 129).

| **Type of surgery (n)** | **Strongly disagree**  (n (%)) | **Disagree**  (n (%)) | **Neutral**  (n (%)) | **Agree**  (n (%)) | **Strongly agree**  (n (%)) |
| --- | --- | --- | --- | --- | --- |
| Leg/hip/ankle/foot (51) | 0 (0.0%) | 5 (9.8%) | 7 (11.9%) | 20 (39.2%) | 22 (43.1%) |
| Abdomen (46) | 4 (8.7%) | 2 (4.3%) | 3 (6.5%) | 15 (32.6%) | 22 (47.8%) |
| Arm/shoulder/hand (11) | 1 (9.1%) | 1 (9.1%) | 1 (9.1%) | 7 (63.6%) | 1 (9.1%) |
| Back (4) | 0 (0.0%) | 1 (25.0%) | 0 (0.0%) | 3 (75.0%) | 0 (0.0%) |
| Groin area (9) | 2 (22.2%) | 4 (44.4%) | 0 (0.0%) | 2 (22.2%) | 1 (11.1%) |
| Head/scalp/face/neck (2) | 0 (0.0%) | 0 (0.0%) | 0 (0.0%) | 1 (50.0%) | 1 (50.0%) |
| Chest (3) | 0 (0.0%) | 0 (0.0%) | 1 (33.3%) | 1 (33.3%) | 1 (33.3%) |
| Abdomen and chest (1) | 0 (0.0%) | 0 (0.0%) | 0 (0.0%) | 1 (100%) | 0 (0.0%) |
| Arm, leg and head (1) | 1 (100%) | 0 (0.0%) | 0 (0.0%) | 0 (0.0%) | 0 (0.0%) |
| Abdomen and groin area (1) | 0 (0.0%) | 0 (0.0%) | 0 (0.0%) | 1 (100%) | 0 (0.0%) |
